# Correction to: Cystic fibrosis–related diabetes onset can be predicted using biomarkers measured at birth

**DOI:** 10.1038/s41436-021-01281-z

**Published:** 2021-08-13

**Authors:** Yu-Chung Lin, Katherine Keenan, Jiafen Gong, Naim Panjwani, Julie Avolio, Fan Lin, Damien Adam, Paula Barrett, Stéphanie Bégin, Yves Berthiaume, Lara Bilodeau, Candice Bjornson, Janna Brusky, Caroline Burgess, Mark Chilvers, Raquel Consunji-Araneta, Guillaume Côté-Maurais, Andrea Dale, Christine Donnelly, Lori Fairservice, Katie Griffin, Natalie Henderson, Angela Hillaby, Daniel Hughes, Shaikh Iqbal, Jennifer Itterman, Mary Jackson, Emma Karlsen, Lorna Kosteniuk, Lynda Lazosky, Winnie Leung, Valerie Levesque, Émilie Maille, Dimas Mateos-Corral, Vanessa McMahon, Mays Merjaneh, Nancy Morrison, Michael Parkins, Jennifer Pike, April Price, Bradley S. Quon, Joe Reisman, Clare Smith, Mary Jane Smith, Nathalie Vadeboncoeur, Danny Veniott, Terry Viczko, Pearce Wilcox, Richard van Wylick, Garry Cutting, Elizabeth Tullis, Felix Ratjen, Johanna M. Rommens, Lei Sun, Melinda Solomon, Anne L. Stephenson, Emmanuelle Brochiero, Scott Blackman, Harriet Corvol, Lisa J. Strug

**Affiliations:** 1 Department of Biostatistics, Dalla Lana School of Public Health, University of Toronto, Toronto, ON Canada; 2 Genetics and Genome Biology, The Hospital for Sick Children, Toronto, ON Canada; 3 Program in Translational Medicine, The Hospital for Sick Children, Toronto, ON Canada; 4 Department of Medicine, Faculty of Medicine, Université de Montréal, Montréal, QC Canada; 5 CRCHUM, Montréal, QC Canada; 6 IWK Health Centre, Halifax, NS Canada; 7 Centre de recherche de l’Institut universitaire de cardiologie et de pneumologie de Québec-Université Laval, Québec City, QC Canada; 8 Alberta Children’s Hospital, Calgary, AB Canada; 9 Jim Pattison Children’s Hospital, Saskatoon, SK Canada; 10 British Columbia Children’s Hospital, Vancouver, BC Canada; 11 The Children’s Hospital of Winnipeg, Winnipeg, MB Canada; 12 Queen Elizabeth II Health Sciences Centre, Halifax, NS Canada; 13 St. Michael’s Hospital, Toronto, ON Canada; 14 Kingston Health Sciences Centre, Kingston, ON Canada; 15 University of Alberta Hospital, Edmonton, AB Canada; 16 The Children’s Hospital of Western Ontario, London, ON Canada; 17 Royal University Hospital, Saskatoon, SK Canada; 18 St. Paul’s Hospital, Vancouver, BC Canada; 19 Foothills Medical Centre, Calgary, AB Canada; 20 The Children’s Hospital of Eastern Ontario, Ottawa, ON Canada; 21 Janeway Children’s Health & Rehabilitation Centre, St. John’s, NL Canada; 22 St. Mary’s General Hospital, Kitchener, ON Canada; 23 McKusick-Nathans Institute of Genetic Medicine, Johns Hopkins University School of Medicine, Baltimore, MD USA; 24 Division of Respiratory Medicine, Hospital for Sick Children, Toronto, ON Canada; 25 Department of Molecular Genetics, University of Toronto, Toronto, ON Canada; 26 Department of Statistical Sciences, University of Toronto, Toronto, ON Canada; 27 Assistance Publique-Hôpitaux de Paris, Hôpital Trousseau, Pediatric Pulmonary Department, Paris, France; 28 Sorbonne Université, Institut National de la Santé et de la Recherche Médicale, Centre de Recherche Saint Antoine, Paris, France; 29 The Center for Applied Genomics, The Hospital for Sick Children, Toronto, ON Canada; 30 Department of Computer Science, University of Toronto, Toronto, ON Canada

Correction to: *Genetics in Medicine* **23**: 927–933; 10.1038/s41436-020-01073-x; Article published online 26 January 2021

The risk alleles of rs1964986 (PRSS1) and rs959173 (CAV1) should be the C allele for both variants instead of the A and T alleles listed in the paper. The changes have been reflected in both Figure 1 and Table 2 shown below.
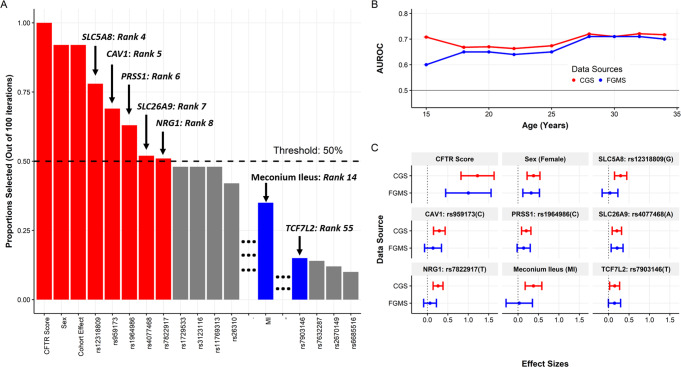


The original article has been corrected.

**Table 2:** Effect sizes (hazard ratios) and the 95% confidence intervals (CIs) fitted using a multivariate Cox PH model in the CGS. Risk allele/risk group noted in parentheses after the listed predictor.

Gene annotation	Predictor	Hazard ratio	95% CI
*CFTR*	*CFTR* mutation score	3.02	(2.01, 4.54)
*—*	Sex (female)	1.48	(1.26, 1.74)
*SLC5A8*	rs12318809 (G)	1.35	(1.16, 1.57)
*CAV1*	rs959173 (C)	1.27	(1.10, 1.47)
*PRSS1*	rs1964986 (C)	1.23	(1.09, 1.38)
*SLC26A9*	rs4077468 (A)	1.20	(1.07, 1.34)
*NRG1*	rs7822917 (T)	1.31	(1.16, 1.48)
*—*	Meconium ileus (MI)	1.29	(1.05, 1.59)
*TCF7L2*	rs7903146 (T)	1.18	(1.05, 1.34)

